# Machine learning for the micropeptide encoded by LINC02381 regulates ferroptosis through the glucose transporter SLC2A10 in glioblastoma

**DOI:** 10.1186/s12885-022-09972-9

**Published:** 2022-08-12

**Authors:** Lan Jiang, Jianke Yang, Qiancheng Xu, Kun Lv, Yunpeng Cao

**Affiliations:** 1 Key Laboratory of Non-Coding RNA Transformation Research of Anhui Higher Education Institution, Yijishan Hospital of Wannan Medical College, Wuhu, China; 2 Central Laboratory, Yijishan Hospital of Wannan Medical College, Wuhu, China; 3 Anhui Provincial Clinical Research Center for Critical Respiratory Disease, Wuhu, China; 4 School of Preclinical Medicine, Wannan Medical College, Wuhu, China; 5 Wuhan Botanical Garden, Chinese Academy of Sciences, Wuhan, 430074 China

**Keywords:** Micropeptide, Glioblastoma, SLC2A10, LINC02381, NRF2

## Abstract

Glioblastoma (GBM) is the most common primary intracranial tumor in the central nervous system, and resistance to temozolomide is an important reason for the failure of GBM treatment. We screened out that Solute Carrier Family 2 Member 10 (SLC2A10) is significantly highly expressed in GBM with a poor prognosis, which is also enriched in the NF-E2 p45-related factor 2 (NRF2) signalling pathway. The NRF2 signalling pathway is an important defence mechanism against ferroptosis. SLC2A10 related LINC02381 is highly expressed in GBM, which is localized in the cytoplasm/exosomes, and LINC02381 encoded micropeptides are localized in the exosomes. The micropeptide encoded by LINC02381 may be a potential treatment strategy for GBM, but the underlying mechanism of its function is not precise yet. We put forward the hypothesis: “The micropeptide encoded by LINC02381 regulates ferroptosis through the glucose transporter SLC2A10 in GBM.” This study innovatively used machine learning for micropeptide to provide personalized diagnosis and treatment plans for precise treatment of GBM, thereby promoting the development of translational medicine. The study aimed to help find new disease diagnoses and prognostic biomarkers and provide a new strategy for experimental scientists to design the downstream validation experiments.

**Supplementary Information:**

The online version contains supplementary material available at 10.1186/s12885-022-09972-9.

## Key points

- We screened out that SLC2A10 was significantly highly expressed in GBM with a poor prognosis, also enriched in the NRF2 signalling pathway.
- SLC2A10 related LINC02381 is highly expressed in GBM, which is localized in the cytoplasm/exosomes, and LINC02381 encoded micropeptides are localized in the exosomes.
- We put forward the hypothesis: “The micropeptide encoded by LINC02381 regulates ferroptosis through the glucose transporter SLC2A10 in GBM.”
- The study aimed to find new disease diagnoses and prognostic biomarkers and provide a new strategy for experimental scientists to design the downstream validation experiments.

## Background

Cancer research is increasingly developing into a serious public health problem worldwide. The pan-cancer analysis of the whole genome (PCAWG) project conducted the most comprehensive tumor genome analysis of the entire genome of 2658 tumors in 38 tumor types [1], comparing the similarities between genome and cell changes found in different tumor types. Gliomas are the most common, and invasive primary intracranial tumor in the central nervous system [2], including adult-type diffuse gliomas, pediatric-type diffuse low-grade gliomas, pediatric-type diffuse high-grade gliomas and circumscribed astrocytic gliomas in the 2021 World Health Organization Classification of tumors of the Central Nervous System (fifth edition) [3]. Glioblastoma (GBM) is a malignant tumor with standard surgery, medication and radiotherapy [4], which belongs to the type of adult-type diffuse gliomas [3].

Multidrug resistance, especially to Temozolomide (TMZ), causing tumors to recur frequently, is a challenge in the treatment of glioblastoma, and its underlying molecular mechanisms are unclear. Since the protection of the blood–brain barrier (BBB) prevents charged or macromolecules from accumulating at physically relevant concentrations in the tumor microenvironment, thus playing the role of a hemolytic tumor, the level of TMZ in the brain is only 40% of the blood content, so it is necessary to develop new treatments that enable the drug to pass through the BBB more effectively to prolong the patient’s survival time beyond the median survival of 15 months [4]. Therefore, it is urgent to explore the process of gliomas’ molecular mechanism and seek new disease diagnoses and prognostic biomarkers. Various researchers have used machine learning to prove the oncological statistical studies, especially cancer prediction and prognosis [5]. For example, machine learning techniques based on the application of MRI derived radiomics to differentiate glioblastoma [6]; a machine learning model integrated the multi-omics data to predict breast cancer survival and progression [7].

We have been working on basic research on gliomas and anti-tumor medicinal plants, including gene clusters, tumor markers [8], and noncoding RNA-based translational medicine research [9]. For example, we identified the potential role of SRY-Box Transcription Factor 6 (SOX6) in GBM [10]; and analyzed the gene set of mitochondrial metabolism-related genes and discovered the importance of Annexin A2 (ANXA2), S100 Calcium Binding Protein A11 (S100A11), and Tubulin Beta 6 Class V (TUBB6) as prognostic factors for GBM [11]. We found the function of sugar transporters (SWEETs) in camellia flower development in the early tea tree [12], while the sugar transporter homologous to humans and plants is Solute Carrier Family 50 Member 1 (SLC50A1). Knowledge of the expression and function of glucose transporters on the human cerebral and cerebral barrier is essential to predict the penetration of the drug BBB and to design strategies to improve drug delivery to intracranial tumors, with significantly lower Solute Carrier Family 2 Member 1 (SLC2A1) protein levels and the Solute Carrier Family 2 Member 3 (SLC2A3) remaining unchanged in GBM microvascular target proteomics [13]. SLC2A1 is a tempting target for transmitting brain-derived neurotrophic factors across the BBB [14].

Micropeptides are small open reading box-coded small peptides [15], less than 100–150 amino acids (A.A.) in length, also known as microproteins or short empty reading frames (sORF-encoded peptides), can also be named after their genomic location, which has been shown to act on maintaining cell stability [16]. Noncoding RNA (circRNA, lncRNA, and pri-miRNA) has a potentially short open reading box that can encode micropeptides, and noncoding RNA-coded short peptides are closely related to tumors. They can be potential prognostic markers and therapeutic targets for tumors. (1) pri-miRNA: through the identification and functional characterization of microprotein miPEP133 encoded in miR-34a primary transcription, it was found that miPEP133 is a tumor inhibitor located in mitochondria that can be used as a potential prognostic marker and therapeutic target for many types of tumors [17]; (2) lncRNA: LINC00998 encoded micropeptide SMIM30 high expression in liver cancer tissue is associated with poor survival rates in patients with liver cancer [18]. SMIM30 promotes the proliferation and migration of liver cancer cells both inside and outside the body. The SMIM30 has a secondary structure of ɑ-helix and is an important adapter for membrane anchoring, and activation of tyrosine kinase SRC/YES1, which activates the MAPK signalling pathway and promotes the development of liver cancer; (3) circRNA: Circ-AKT3 encoded AKT3-174aa through overlapping starting and terminating cipher subcodes. Overexpression of AKT3-174aa could reduce the cell proliferation of GBM, AKT3-174aa plays a negative regulatory role in the PI3K/AKT signalling pathway [19].

Ferroptosis is a regulated cell death caused by excessive lipid peroxidation, catalyzed by iron ions [20], which is associated with various diseases and has been found to intervene in the development of disease by activating or inhibiting ferroptosis. Many oxidation and antioxidant systems work together with autophagy and membrane repair mechanisms to form a lipid peroxidation process during ferroptosis [21]. Ferroptosis plays a dual role in tumor promotion and inhibition, depending on the release of the molecular patterns associated with damage in the tumor microenvironment and the activation of the immune response caused by ferroptosis injury in the tumor occurrence [22]. Ferroptosis affects the efficacy of chemotherapy, radiotherapy, and immunotherapy, so combined with drugs that target ferroptosis signalling could improve the outcome of these treatments.

## Materials and methods

### Screening of gene sets of SWEETs

The human-based SWEETs are filtered with the Molecular Signatures Database (MSigDB) (https://www.gsea-msigdb.org/gsea/msigdb/search.jsp) and WikiGene [23] databases. We searched the PubMed [24] database to collect the progress of SWEETs in tumors and GBM.

Using the STRING v11.5 (https://string-db.org) online platform to build the SWEETs Protein–protein interaction (PPI) network, we also obtained the protein information and combined functional enrichment analysis (Kyoto Encyclopedia of Genes and Genomes (KEGG) / Reactome pathway and Gene Ontology (G.O.) analysis).

### The significant difference in expression and survival analysis of SWEETs in pan-cancer

We downloaded the expressional and clinical data from TCGA pan-cancer (https://portal.gdc.cancer.gov), GEO (GSE51024) and Genotype-Tissue Expression (GTEx) database, and extracted data from tumor and normal tissue. After removing the batch effects, we calculated the differentially expressed genes (DEGs) (|logFC|> 1, adjusted p-value < 0.05) by limma package [25], and extracted the differentially expressed SWEETs (DE-SWEETs) in pan-cancer. The overall survival (OS) of SWEETs-related patients was calculated by cox method (p-value < 0.05) [11]. Drawing the Sanger plot to show the significant correlation between DE-SWEETs with O.S. analysis and pan-cancer. We also screened both DE-SWEETs and the high risk of poor prognosis in multi-cancers by the Sanger plot.

### Verification and ceRNA prediction of SLC2A10 in glioblastoma

SLC2A10-related ceRNA prediction was performed to screen for crucial lncRNA in combination with lnCeVar [26] and multiMiR [27]. SLC2A10 and LINC02381 were verified using GEPIA2 [28]/EMBL-EBI [29] /GEO [30] to demonstrate the significant differences in the representation of GBM compared with normal tissues in multiple databases.

### Micropeptide identification and functional analysis

We used MiPepid to predict the LINC02381 encoded micropeptide (> 0.99) [31] and the RIdeogram to draw the predicted micropeptides on human chromosomes [32]. The identification and functional enrichment analyses of predicted micropeptides were carried out using smORFunction [33].

SnapGene Viewer 5.2.3 (http://www.snapgene.com/) was used to present G.C. content and location information and sequence information for LINC02381 and its encoded micropeptides, and iLoc-LncRNA [34] was used to predict the location of LINC02381 and its encoded micropeptides.

## Results

**Fig. 1:**
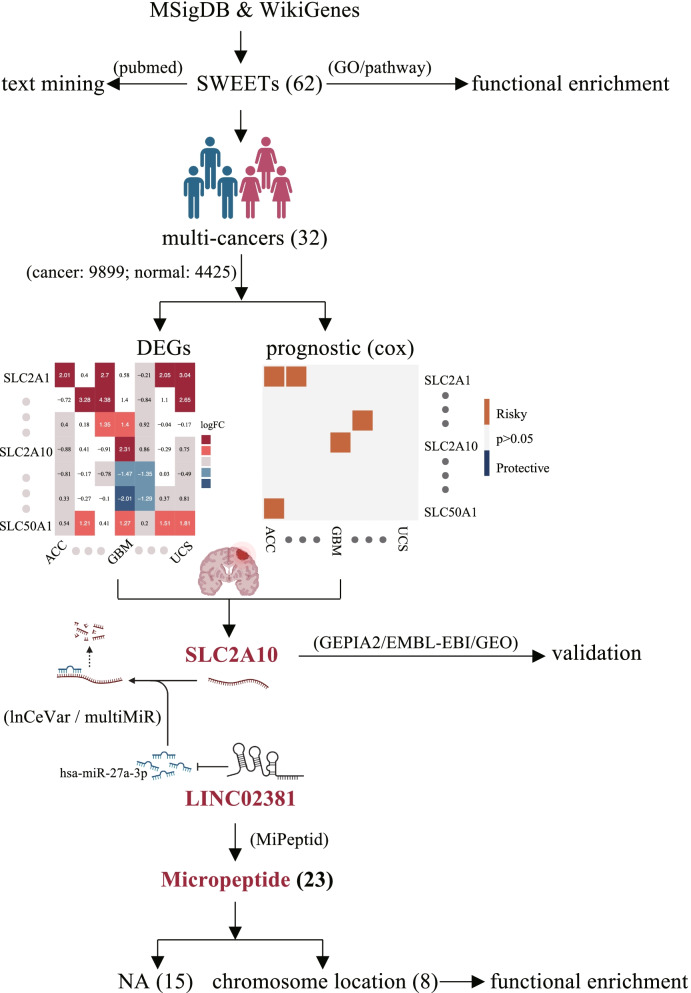
The integrated workflow to perform the strategies for finding the emerging role of SWEETs related micropeptides in GBM

We provided the integrated workflow to perform the strategies for finding the emerging role of SWEETs related to micropeptides in GBM (Fig. 1).

### SWEETs screening, literature research and functional enrichment analysis

We screened 62 human-derived SWEETs in 5 categories: facilitative glucose transporter, glucose cotransporter, nucleotide-sugar transporter, sugar-phosphate/phosphate exchanger and sugar efflux transporter of the SWEET family through the MSigDB and WiKigene databases.

**Fig. 2:**
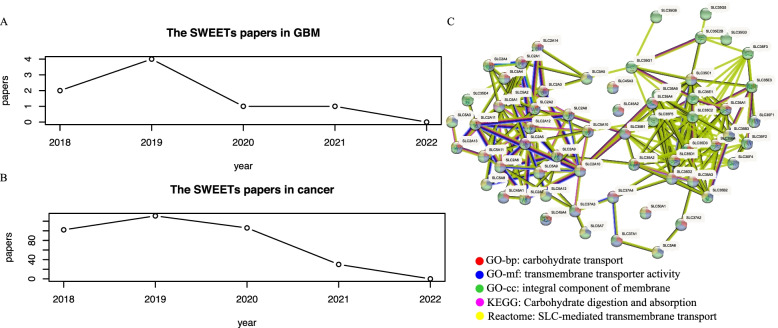
Text mining and functional enrichment analysis of the SWEETs. Text mining in **A** GBM or **B** cancer from the PubMed database. **C** PPI and available enrichment analysis

The text mining of PubMed illustrated the research progress of SWEETs in GBM and cancers (Fig. 2A and B) in the last four years (2018–2021). It was found that there were only four papers in GBM and just over 120 papers in 2019, and more researchers need to study them in-depth. We performed the STRING online database’s PPI network analysis and functional enrichment analysis (Fig. 2C). G.O. results revealed that “carbohydrate transport” (GO-biological process (GO-bp)), “transmembrane transporter activity” (GO-molecular function (GO-mf)), and “integral component of membrane” (GO-cellular component (GO-cc)). SWEETs are also enriched in “carbohydrate digestion and absorption” (KEGG) and “SLC-mediated transmembrane transport” (Reactome).

### Difference expression and prognosis analysis of SWEETs in pan-cancer

**Fig. 3:**
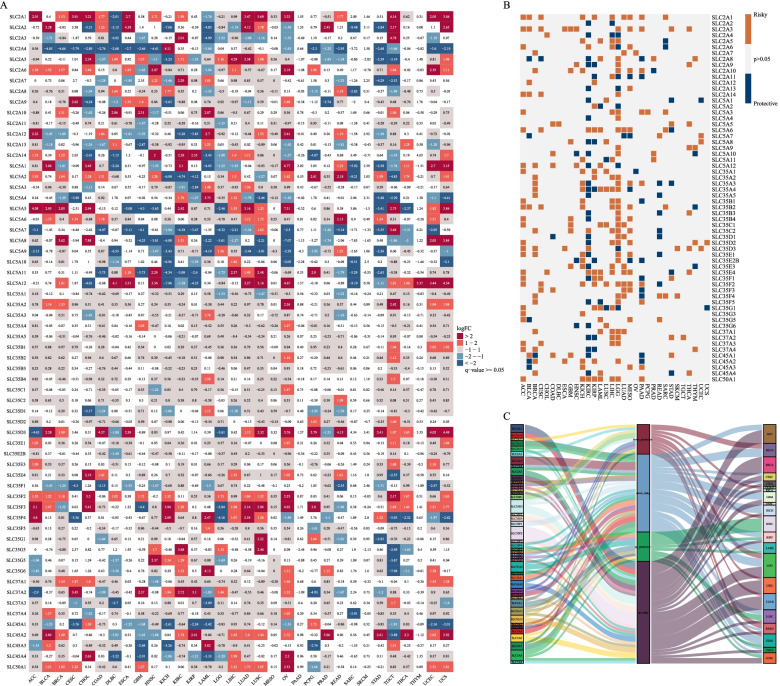
Differentially expressed and survival analysis of SWEETs in multi-cancers. **A** DE-SWEETs in 32 types of cancers. **B** Summary of the correlation between expression of glucose transporters and patient survival. Orange represents a higher expression of glucose transporters associated with worse survival, and blue represents an association with better survival. Only p-value < 0.05 are shown. **C** Sankey plot of DE-SWEETs with survival analysis in multi-cancers

We downloaded the pan-cancer data on tumor tissue and normal tissue from the public database TCGA, GEO and GTEx, for a total of 32 tumors, of which 9,989 were tumor tissue, and 4,425 were normal tissue (Table S 11). We found that SWEETs were significantly highly expressed in many tumors (Fig. 3A), and screened the prognosis analysis of SWEETs in pan-cancer (Fig. 3B). DE-SWEETs with survival analysis in multi-cancers were performed by sanger plot (Fig. 3C).

**Fig. 4:**
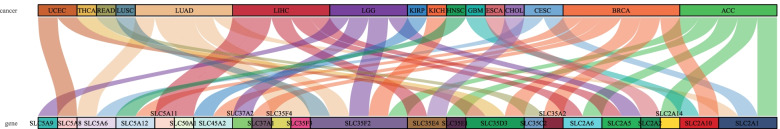
Sankey plot of DE-SWEETs with worse survival in multi-cancers

To give a more evident plot of the response of significantly highly expressed with poorly prognostic DE-SWEETs in pan-cancer, we redrawn the sanger plot and found that only SLC2A10 was significantly highly expressed with poor prognosis in GBM (Fig. 4).

**Fig. 5:**
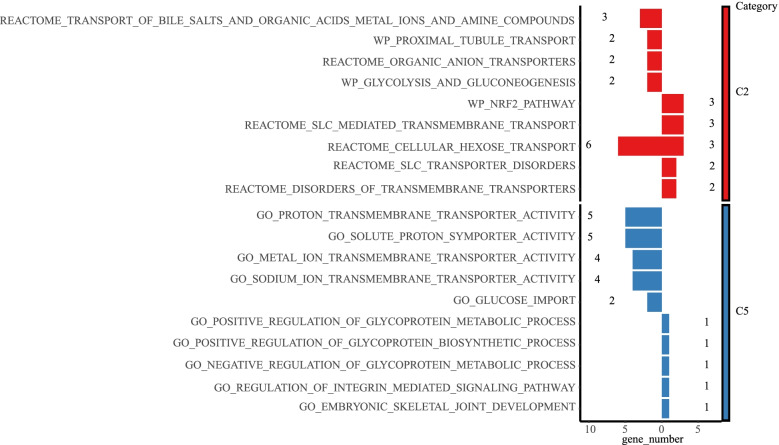
Functional enrichment analysis

DE-SWEETs in GBM were combined with the c2.cp.kegg.v7.entrez.gmt and c5.go.v7.entrez.gmt in MSigDB to conduct enrichment analysis. We predicted its potential function in GBM (Fig. 5), c2 (red) indicated pathway enrichment results, and c5(blue) represented the enrichment results of G.O. annotations. The histograms indicated down-regulated DE-SWEETs to the left and up-regulated DE-SWEETs to the right. We observed that the significantly high expression of SWEETs (SLC2A10/SLC2A5/SLC2A9) in GBM was significantly concentrated in the WikiPathway database in the classic NRF2 signalling pathway, which was closely related to the regulation of ferroptosis.

### CeRNA prediction and target lncRNA determination

**Fig. 6:**
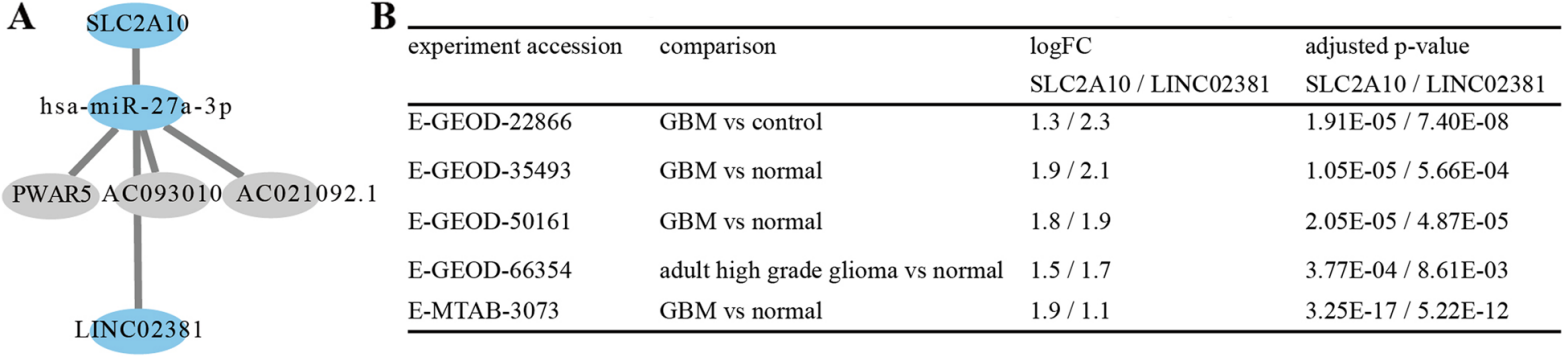
CeRNA prediction and expressed validation, **A** ceRNA prediction and **B** validation dataset for SLC2A10 and LINC02381

We screened out SLC2A10, a key target in GBM, further obtained the verified relevant ceRNA mechanism by multiMiR and found that four lncRNAs might be related to SLC2A10 (Fig. 6A). Subsequently, after predicting the differential expression of lncRNA in GBM, we found that LINC02381 was significantly highly expressed in GBM, LINC02381/hsa-miR-27a-3p/SLC2A10 axis had the potential ceRNA mechanism in GBM. The expressions of SLC2A10 and LINC02381 were verified by the external validation dataset (Fig. 6B).

### Identification of lncRNA-coded micropeptides and prediction of their function

**Fig. 7:**
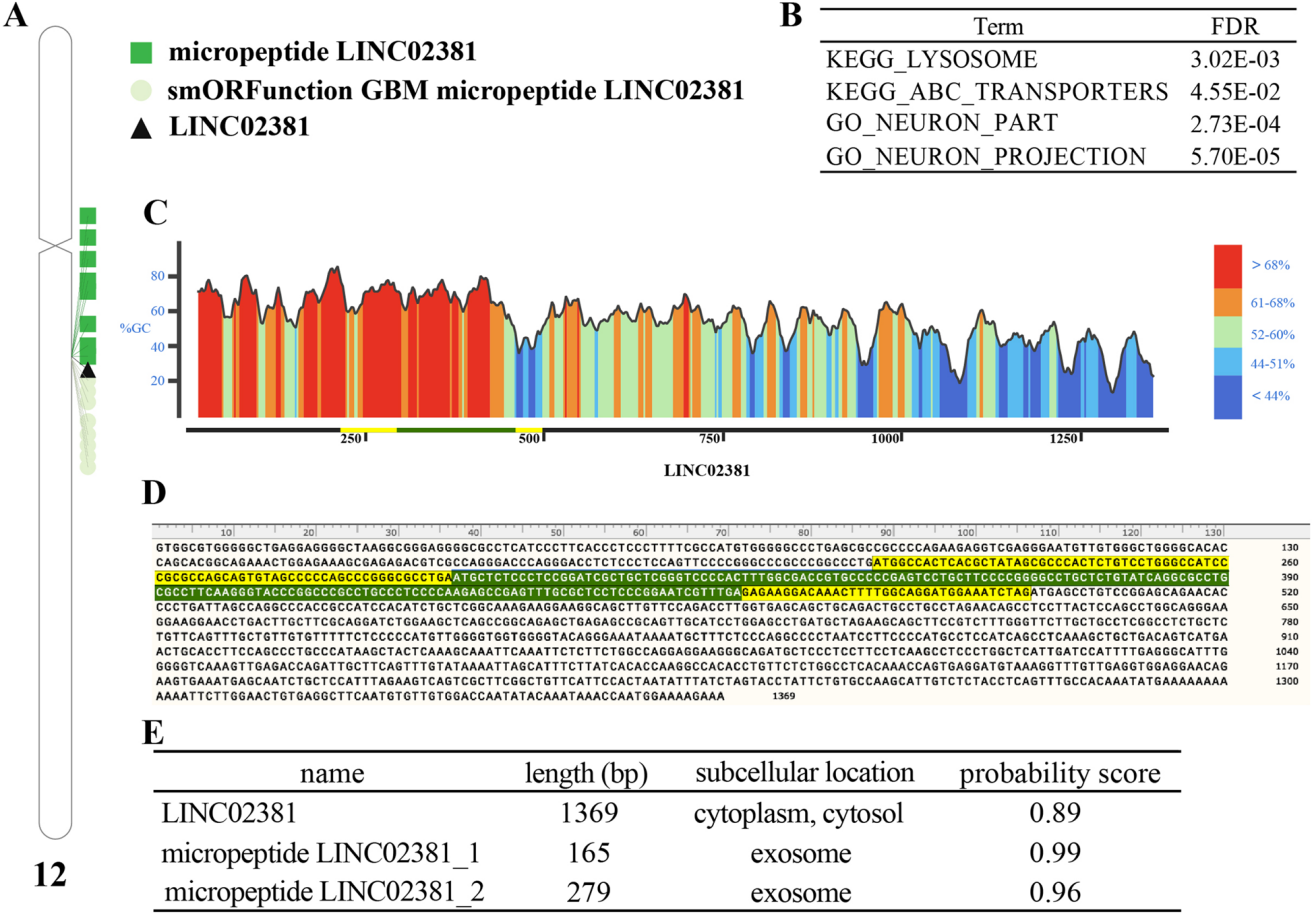
LINC02381 encoded micropeptide prediction. **A** chromosome location, **B** the functional enrichment analysis of micropeptide in GBM, **C** G.C. content of LINC02381 and micropeptide, **D** visualization of sequence, green means micropeptide LINC02381_1, yellow means micropeptide LINC02381_2, **E** subcellular location prediction

After locking in the critical LINC02381, we tried MiPepid to predict LINC02381-aa and used smORFunction to identify and functionally analyze the predicted micropeptides (Fig. 7). The chromosome map showed that LINC02381 and its encoded micropeptides were located on the human chromosome 12, and we identified that smORF with potential function in GBM was also located on chromosome 12 (Fig. 7A). LINC02381-aa might be associated with ABC transporters, neuronal parts and projection (Fig. 7B) in GBM. We then used SnapGene Viewer 5.2.3 to present G.C. content and location information (Fig. 7C) and sequence information (Fig. 7D) for LINC02381 and its encoded micropeptides. We found that LINC02381 was located in the cytoplasm, and its encoded micropeptides were located in the exosome by iLoc-LncRNA (Fig. 7E).

## Discussion

Knowledge of the expression and function of glucose transporters on the human cerebral and cerebral barrier is essential to predict the penetration of the drug BBB and to design strategies to improve drug delivery to intracranial tumors, with significantly lower levels of SLC2A1 protein and the same in SLC2A3 in GBM microvascular-targeted proteomics [13]. SLC2A1 is a compelling target for transmitting brain-derived neurotrophic factors across the BBB [14]. SLC2A3 has a poor prognosis in GBM, is significantly increased in cell lines treated with TMZ and DIDIT4 overexpression, and DDIT4 mediates the expression of SLC2A3 and reduces the toxicity of TMZ. The ATF4/DDIT4 signal is associated with the dryness of GBM autophagy and SLC2A3 regulation, TMZ resistance and poor prognosis in GBM patients, and the targeted DDIT4/SLC2A3 signal may be the new direction of treatment for GBM [35]. SLC2A4 inhibitors reduce glucose consumption and lactic acid products and inhibit GBM U87MG and Hu197 cell proliferation [36]. SLC2A5 was expressed in GBM [37]. We believe that studying the gene family of human-derived SWEETs may enable drugs to pass through the BBB more effectively. We initially screened the human-derived SWEETs through MSigDB and WikiGene. We selected a total of 62 in 5 categories: facilitative GLUT transporter, sodium-glucose cotransporter, nucleotide-sugar transporter, sugar-phosphate/phosphate exchanger and sugar efflux transporters of the SWEET family.

In combination with the PubMed literature search for nearly four years (2018–2021), the progress of glucose transporters in tumors and predict its potential function in tumors. The differential expression and prognosis relationship between pan-cancers are then clarified. In the case of glioblastoma, SWEETs expressed with significant differences were combined with c2.cp.kegg.v7.2.entrez.gmt and c5.go.v7.entrez.gmt, and found that the significantly highly expression of SWEETs (SLC2A10/SLC2A5/SLC2A9) in the WikiPathway database is significantly concentrated in the NRF2 signalling pathway. The NRF2 signalling pathway regulates glutathione stability, mitochondrial function, and lipid metabolism and is associated with ferroptosis.

We screened out the key target SLC2A10 in GBM, further obtained the verified ceRNA mechanism through multiMiR, and found that LINC02381 was significantly highly expressed in GBM, and confirmed the expression of SLC2A10 and LINC02381 in GBM, LINC02381/hsa-miR-27a-3p/SLC2A10 has a potential ceRNA mechanism in GBM. At this stage, there is no study of the glucose transporter SLC2A10 and its associated LINC02381 in GBM. LncRNA and pri-miRNA were not involved in the noncoding RNA encoded micropeptide in GBM. Previous studies have revealed that circRNA-coded micropeptide AKT3-174aa, FBXW7-185aa, SHPRH-146aa, and PINT-87aa might be independent prognostic indicators and therapeutic targets in GBM [38]. Whether lncRNA-encoded micropeptides in glioblastoma are new prognostic or diagnostic indicators requires further exploration. After locking in the vital LINC02381, we used MiPepid to predict the LINC02381 encoded micropeptides and visualized them from chromosomal positioning, functional analysis, and G.C. content, sequence information, subcellular position and initially named the LINC02381 encoded micropeptides as LINC02381-aa. Through subcellular position prediction, LINC02381 was found to be located in the cytoplasm whereas LINC02381-aa in exosomes.

Noncoding RNA is abundant and stable in the bioactive ingredients of exosomes, which could regulate cell proliferation, invasion, angiogenesis, immune escape, and therapeutic resistance in GBM. The excellent biocompatibility of exosomes could also be transformed into therapeutic factor-targeted delivery systems to improve the accuracy and efficacy of drug treatment. Exosomes are expected to become new biomarkers and therapeutic agents for GBM, and explicitly targeting exosome noncoding RNA is a potential GBM treatment strategy with potential [39]. However, micropeptides encoded by exosome lncRNA have not been studied in GBM yet.

**Fig. 8:**
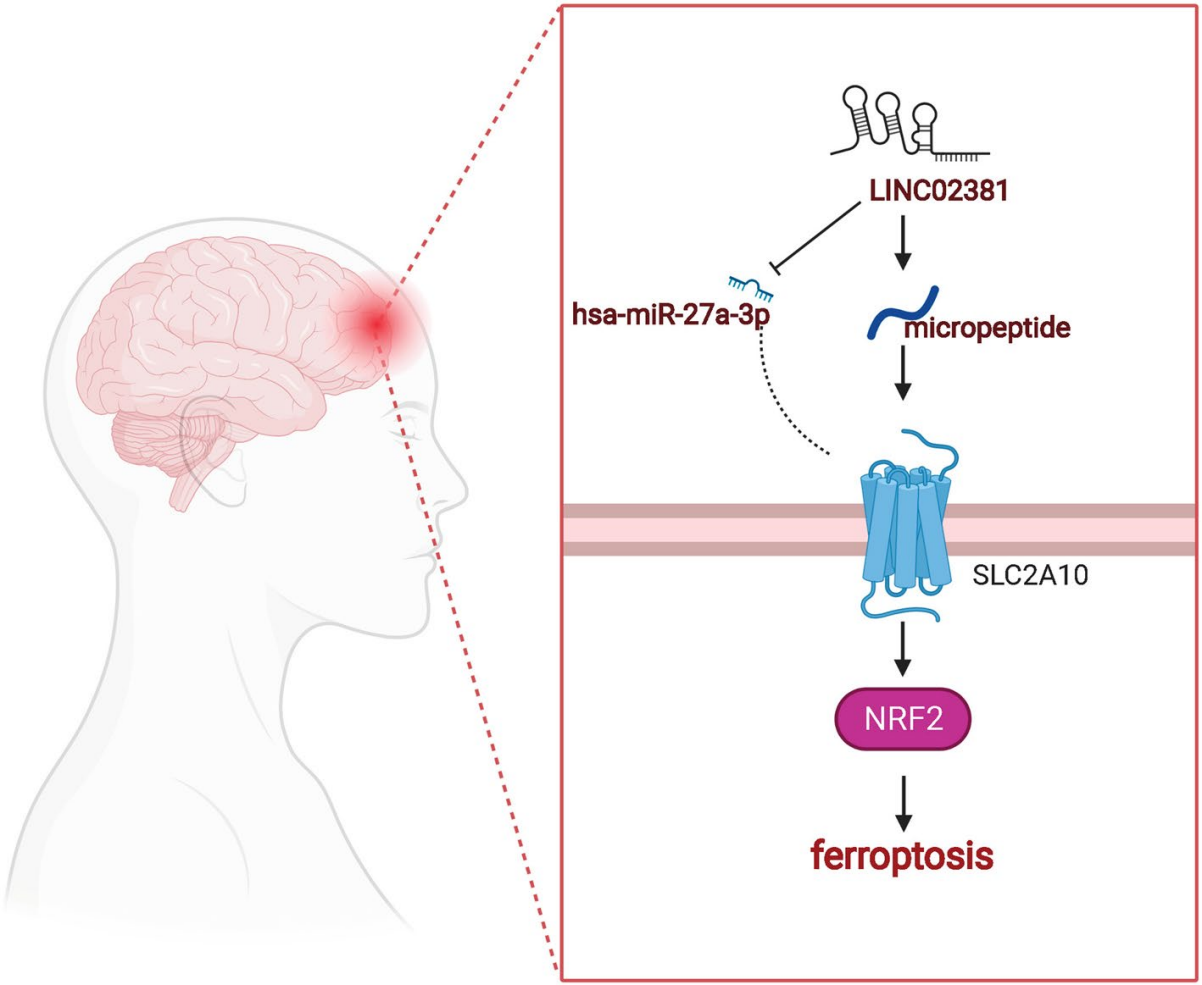
The hypothesis diagram: LINC02381 encoded micropeptides regulate ferroptosis through SLC2A10 in GBM

NRF2 is a transcription factor that regulates cell defence and resists oxidative stress and plays a dual role in tumor progression [40]. Many components of the ferroptosis cascade reaction are targeted at NRF2, indicating that NRF2 mediates ferroptosis response, NRF2 is a critical gene for ferroptosis, looking for treatment for ferroptosis-driven [41]. The genetic regulation of NRF2 expression, including knock-down and overexpression, can fine-tune the sensitivity of glioma cells to ferroptosis inducers erastin and RSL3 [42]. The NRF2 signal pathway is an important defence mechanism against ferroptosis. According to the previous studies, we believe that: SLC2A10 in GBM had significantly highly expression and poor prognosis, significantly concentrated in the NRF2 signalling pathway. SLC2A10-related LINC02381 is also significantly highly expressed in GBM, which is located in cytoplasm/exosomes, and its encoded micropeptides are found in exosomes. Previously reported have revealed that knock-down LINC02381 expression in GBM cells and assessed their viability and clonogenic ability [43]. LINC02381 was up-regulated in glioma cells by RT-qPCR and facilitated glioma cell proliferation and suppressed cell apoptosis via a knockdown experiment [43]. LINC02381 promoted glioma cell growth via CBX5 by rescue experiment [43]. The NRF2 signalling pathway is an important defence mechanism against ferroptosis. LINC02381-aa might be a potential GBM treatment strategy, but the underlying mechanisms for its effects are unclear. Therefore, we propose the following hypothesis: LINC02381 encoded micropeptides regulate ferroptosis through SLC2A10 in GBM (Fig. 8).

## Conclusions

We screened 62 SWEETs in pan-cancer and found that they highly expressed with worse survival SLC2A10 as a critical biomarker in GBM and related to the NRF2 signalling pathway. We further obtained the verified relevant ceRNA mechanism, LINC02381/hsa-miR-27a-3p/SLC2A10. SLC2A10-related LINC02381 was also significantly highly expressed in GBM, which is located in cytoplasm/exosomes, and its encoded micropeptides were located in exosomes. Therefore, we propose the following hypothesis: LINC02381 encoded micropeptides regulate ferroptosis through SLC2A10 in GBM. The study aimed to find new disease diagnoses and prognostic biomarkers and provide a new strategy for experimental scientists to design the downstream validation experiments.

## Supplementary Information

**Additional file 1: Table S1.** The list of pan-cancer in this study.

## Data Availability

Origin data was uploaded to JianguoYun. Below is the link: https://www.jianguoyun.com/p/DTWtKosQ7tTfCRjlxJME

## Abbreviations

GBM: Glioblastoma
SLC2A10: Solute Carrier Family 2 Member 10
NRF2: NF-E2 p45-related factor 2
PCAWG: Pan-cancer analysis of the whole genome
TMZ: Temozolomide
BBB: Blood-brain barrier
SOX6: SRY-Box Transcription Factor 6
ANXA2: Annexin A2
SWEETs: Sugar transporters
S100A11: S100 Calcium Binding Protein A11
TUBB6: Tubulin Beta 6 Class V
SLC50A1: Solute Carrier Family 50 Member 1
SLC2A1: Solute Carrier Family 2 Member 1
SLC2A3: Solute Carrier Family 2 Member 3
A.A.: Amino acids
sORF-encoded peptides: Short open reading frames
MSigDB: Molecular Signatures Database
PPI: Protein-protein interaction
KEGG: Kyoto Encyclopedia of Genes and Genomes
G.O.: Gene Ontology
GTEx: Genotype-Tissue Expression
DEGs: Differentially expressed genes
DE-SWEETs: Differentially expressed SWEETs
O.S.: Overall survival
GO-bp: GO-biological process
GO-mf: GO-molecular function
GO-cc: GO-cellular component
